# Association of *Helicobacter pylori babA2* gene and gastric cancer risk: a meta-analysis

**DOI:** 10.1186/s12885-020-06962-7

**Published:** 2020-05-24

**Authors:** Marce-Amara Kpoghomou, Jinchen Wang, Tianpei Wang, Guanfu Jin

**Affiliations:** 1 Department of Epidemiology, Center for Global Health, School of Public Health, Nanjing Medical University, Nanjing, 211166 China; 2 Jiangsu Key Lab of Cancer Biomarkers, Prevention and Treatment, Collaborative Innovation Center for Cancer Medicine, Nanjing Medical University, Nanjing, 211166 China

**Keywords:** *Helicobacter pylori*, *babA2* gene, Gastric cancer, Meta-analysis

## Abstract

**Background:**

The association of *Helicobacter pylori* (*H. pylori*) *babA2* gene with gastric cancer (GC) was reported by several studies, but results were inconsistent. This meta-analysis was performed to investigate the relationship between *H. pylori babA2* gene and GC risk.

**Methods:**

Case-control studies involving the association between *H. pylori babA2* gene and GC risk were systematically identified from PubMed databases. A meta-analysis was used to pool studies and to estimate odds ratios (ORs) with 95% confidence intervals (CIs) of *H. pylori babA2* gene associated with GC risk.

**Results:**

Twenty studies were identified with a total of 1289 GC cases and 1081 controls. *H. pylori babA2* gene was associated with an increased risk of GC by 2.05 fold (95% CI, 1.30–3.24, *P* = 0.002*)*. In subgroup analysis, we found that *H. pylori babA2* gene was significantly associated with GC risk in Asian population (OR = 2.63, 95% CI: 1.36–5.09 *P* = 0.004) but not in South American population (OR = 1.35, 95% CI: 0.69–2.64, *P = 0.379*).

**Conclusions:**

This meta-analysis indicates that *H. pylori babA2* gene may be associated with increased risk of GC, especially in Asian population.

## Background

Gastric Cancer (GC) is the fifth most common cancer and the third leading cause of mortality worldwide [1–3], with approximately 42.5% of all cases diagnosed in China [4, 5]. About 1 million incident cases of GC are annually projected, with the majority observed in Eastern Asia, Latin America and Eastern Europe [5]. In 2015, GC was the second most common cancer with about 6,791,000 new cases in China [4]. Genetic and environment factors are involved in GC development. *H. pylori* infection, cigarette smoking, low intake of fresh vegetables and fruits and salty foods are main risk factors of GC [6].

*Helicobacter pylori* (*H. pylori*) infection is the most common human infections inhabiting in the stomach. It is a gram-negative bacterium, which has epigenetic effects on gastric epithelial cells and indirect inflammatory response on the gastric mucosa [7]. Some studies showed that *H. pylori* alone or associated gene were strongly associated with gastric cancer risk [8–10]. According to the International Agency for Research on Cancer, *H. pylori* was defined as a class I carcinogen [11]. However, only a small fraction of infected patients develop severe diseases [12]. *H. pylori* is well distinguished to have a high level of genetic variations allowing it to be adapted to the host gastric epithelium [13]. It is well described that different strains of *H. pylori* showed different degrees of virulence [14–16]. *H. pylori* strains harboring the cytotoxin-associated antigen (*cagA*) and the vacuolating toxin A (*vacA*) have been considered as risk factors for GC [15, 17, 18]. The OipA, one of porin proteins associated with severe neutrophil infiltration in IL-8 induction and gastric colonization [19], was also associated with GC risk [20–22].

The blood-group antigen-binding adhesin (*babA*) encoded by *babA2* gene is a major adhesin on the outer membrane of *H. pylori*. *BabA2* is characterized to be an active gene in the binding activity of Lewis-b blood group antigen on gastric epithelium and host cell and determine *H. pylori* colonization density [23, 24]. The sequence of the three *babA* gene alleles have been identified (*babA1*, *babA2* and *babB*), but only the *babA2* is involve d in Lewis-b binding activity. To date, several studies have evaluated the effect of *H. pylori babA2* gene on risk of GC [19, 25–42], but the results are conflicting possibly due to small sample size of single studies. In the present study, we conducted a meta-analysis to assess the association between *H. pylori babA2* gene and GC risk based on published cases-control studies.

## Methods

### Search strategies

All relevant studies were identified from PubMed databases. The search strategy included the terms (“*babA2*” OR “antigen-binding adhesion gene”) AND (“*Helicobacter pylori*” OR “*H. pylori* infection”) AND (“genotype” OR “polymorphism”) AND (“gastric cancer” OR “stomach cancer”) in any text field of the database. In addition, we also collected additional studies from references of original and review articles.

### Inclusion and exclusion criteria

Inclusion criteria to select studies for this meta-analysis were as follows: (1) study describing the relationship between *H. pylori babA2* gene and GC, (2) studies that provided *babA2* positive frequencies, (3) studies published in English with full text available. Exclusion criteria were as follow: (1) insufficient data to calculate OR and 95% CI, (2) vivo or experimental studies, and (3) meta-analysis or review studies.

### Data extraction

Data were extracted from each study independently by two investigators and contradictions between them were discussed to obtain agreement. The following information’s were collected: first author’s name, year of publication, country, ethnicity, sample size, type of study, source of sample, study quality assessment, *babA2* positive frequencies, OR estimation and 95% CI for the association between *H. pylori babA2* gene and GC.

### Quality score assessment

Quality score of each included study was assessed by the same two authors independently using the Newcastle-Ottawa Quality Assessment Scale (NOS) for case-control studies [43]. The NOS is a validated quality assessment for case-control studies with three parameters for quality: selection, comparability and exposure. The maximum score of each parameter is 4 for selection, 2 for comparability and 3 for exposure.

### Statistical analysis

The pooled ORs with 95% CIs were used to indicate the effect of *H. pylori babA2* gene effect on GC risk. χ^2^ base on *Q* test and *I*^2^ statistics were used to evaluate the statistical heterogeneity among included studies. The fixed-effects model was used when there was no significant heterogeneity (*P* ≥ 0.10 and *I*^*2*^ ≤ 50%) [44, 45] between studies, otherwise the random effect model was applied to provide more conservative estimates [46]. In addition, we performed subgroup analysis by ethnicity and quality score assessment. Ethnicities were divided into Asian, European, South American and North American. Moreover, sensitivity analyses were performed to estimate the effect of each included study on overall effect. We used Begg’s test and Egger’s test to estimate publication bias [47]. All the statistical analyses were performed using STATA 11.0.

## Results

### Characteristics of selected studies

**Fig. 1:**
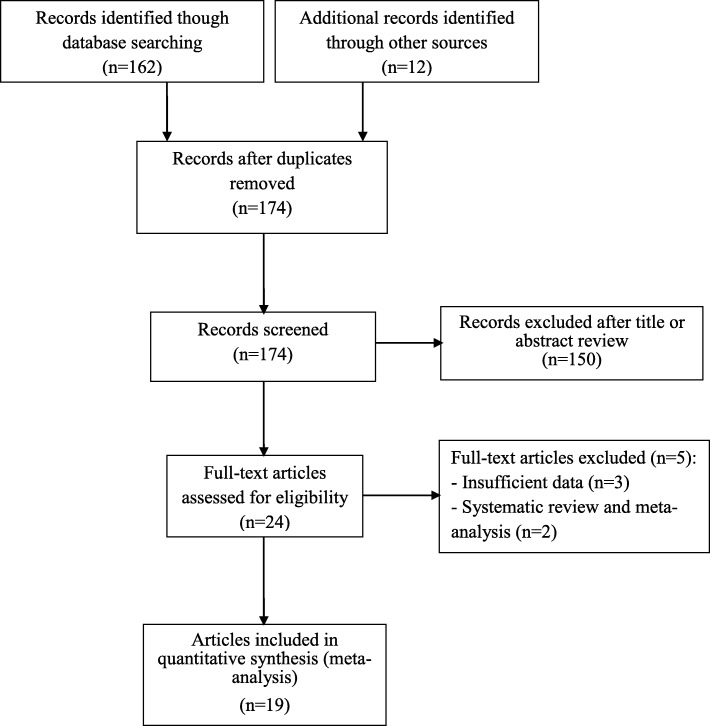
The flowchart of literature search and study inclusion

Literature research strategy is detailed in Fig. 1. There were 174 potentially relevant studies. After title and abstract evaluation, 24 articles with full-text assessment were included when duplicated studies were excluded. After full-text reviewed, a total of 19 eligible articles were included in this meta-analysis, and 5 articles were excluded because of the following reasons: two articles were reviews [48, 49], and three articles had insufficient data [50–52]. One article included participants from two countries [19], which were considered as two independent studies for subsequent data extraction and meta-analysis. Among 20 studies, 5 were from South American population [19, 26, 32, 34, 40], 13 from Asian population [25, 27–31, 33, 35–39, 41], one from North American population [19] and one from European population [42] (Table 1).

**Table 1:** Characteristics of included studies

First Author	Year	Country	Ethnicity	Control	Sample Size (case/control)	OR (95%CI)	Quality Assessment
Gerhard	1999	Germany	European	Gastritis	39/23	3.31 (1.07–10.17)	5
Mizushima	2001	Japan	Asian	NUD	70/12	2.12 (0.58–7.68)	5
Yamaoka	2002	United States	North American	Gastritis	47/23	0.74 (0.27–2.02)	5
Yamaoka	2002	Colombia	South American	Gastritis	62/19	2.08 (0.72–6.00)	5
Oliveira	2003	Brazil	South American	Gastritis	53/75	2.73 (1.32–5.67)	7
Han	2004	Shanghai	Asian	Chronic gastritis	40/24	0.71 (0.25–2.08)	6
Lee	2006	South korea	Asian	Routine gastoscopy	98/136	3.98 (1.94–8.15)	7
Chomvarin	2007	Thailand	Asian	NUG	72/6	1.32 (0.14–12.13)	6
Zhang	2008	China	Asian	Gastritis	143/69	1.07 (0.59–1.94)	6
Erzin	2008	Turkey	Asian	NUD	36/34	31.07 (8.22–117.52)	6
Bartchewsky	2009	Brazil	South American	Gastritis	142/38	0.96 (0.44–2.12)	6
Safaei	2010	Iran	Asian	CAG	38/16	1.87 (0.35–9.96)	5
Mattar	2010	Brazil	South American	Gastritis	36/32	0.38 (0.14–1.02)	5
Saxena	2011	India	Asian	NUD	45/123	1.12 (0.49–2.57)	7
Abadi	2011	Iran	Asian	NUD	55/50	53.65 (11.67–246.69)	5
Mottaghi	2014	Iran	Asian	Chronic gastritis	60/12	0.65 (0.18–2.31)	6
Abdi	2016	Iran	Asian	NAG	22/61	2.81 (1.02–7.74)	6
Roman-Roman	2017	Mexico	South American	Chronic gastritis	109/282	1.93 (0.83–4.50)	7
Heidari	2017	Iran	Asian	Gastritis	32/22	1.12 (0.35–3.57)	5
Bartpho	2020	Thailand	Asian	Chronic gastritis	90/24	7.38 (2.64–20.09)	6

*NUD* Non-ulcer Dyspepsia, *NAG* Non-atrophic gastritis, *CAG* Chronic active gastritis

### Meta-analysis

**Fig. 2:**
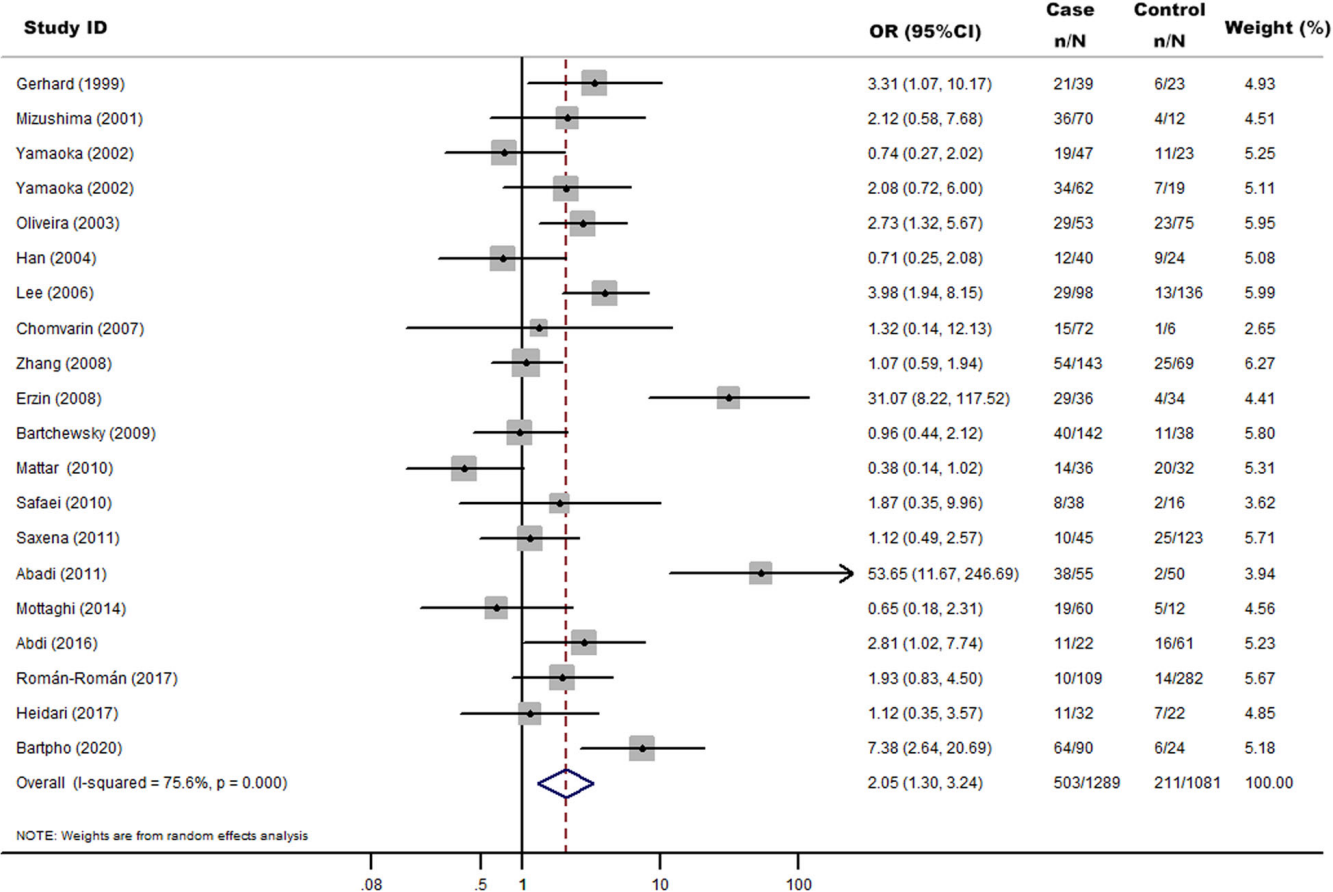
Random-effects meta-analysis forest plot of the odds ratio of gastric cancer according to *H.pylori* infection with *babA2* with respect to without *babA2*. The studies are sorted by publication year. The solid squares are centered on the odds ratio (OR) point estimate from each study, and the horizontal line through each square indicates the 95% confidence interval (CI) for the study. The area of each square represents the magnitude of association, and the horizontal tips of the diamond represent the 95% CI

**Fig. 3:**
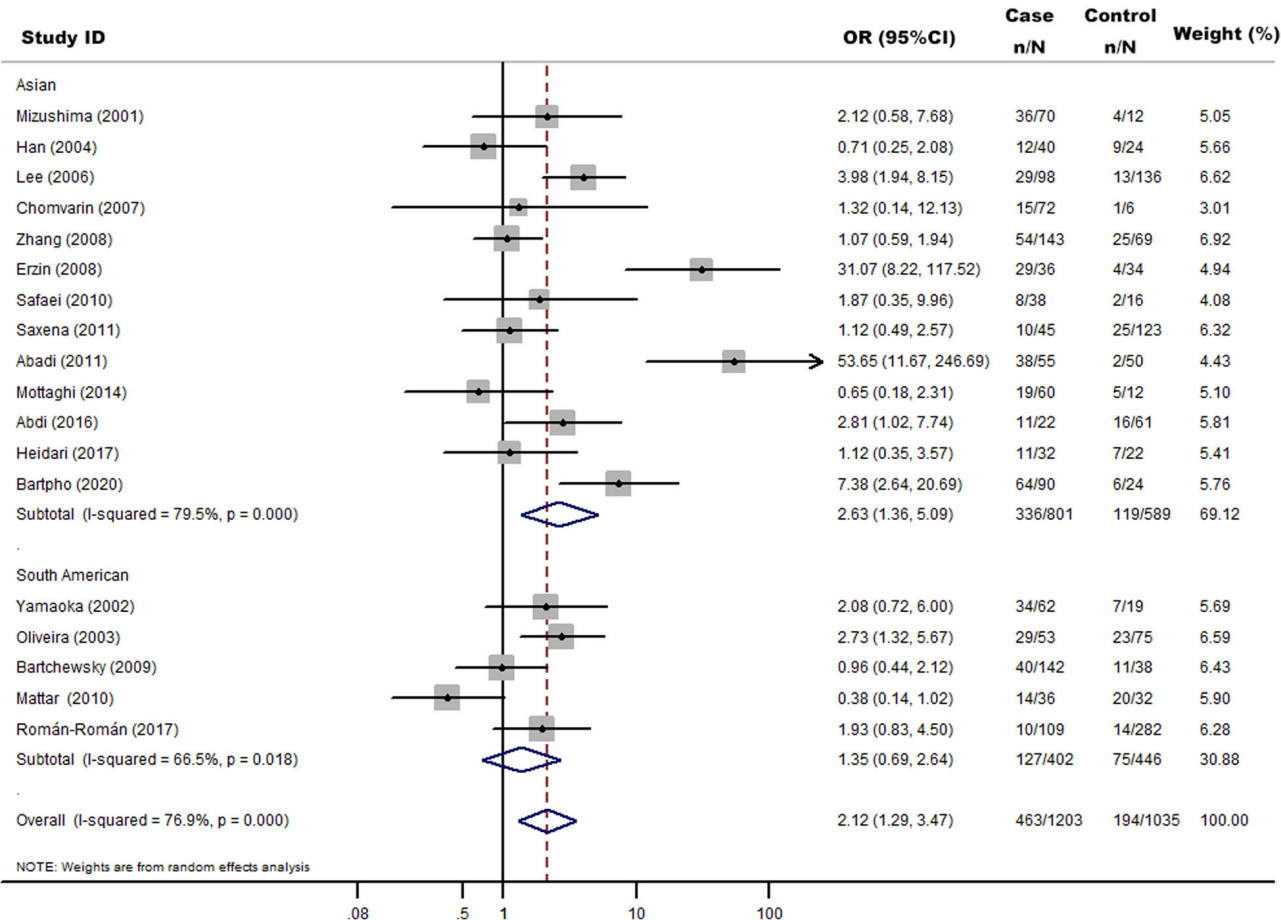
Random-effects meta-analysis forest plot of the odds ratio of gastric cancer according to *H.pylori* infection with *babA2* with respect to without *babA2*. The studies are divided into Asian and South American according to participants ethnicities and sorted by publication year. The solid squares are centered on the odds ratio (OR) point estimate from each study, and the horizontal line through each square indicates the 95% confidence interval (CI) for the study. The area of each square represents the magnitude of association, and the horizontal tips of the diamond represent the 95% CI

There were 20 studies [19, 25–42] that investigated the association between *H. pylori babA2* gene and GC risk. In total, 1289 cases and 1081 controls were included in this meta-analysis (Table 1). The overall proportions of *H. pylori babA2* were 39.02% (503/1289) in GC cases and 19.52% (211/1081) in controls. *H. pylori babA2* gene was significantly associated with an increased risk of GC (OR = 2.05, 95% CI: 1.30–3.24, *P* = 0.002) (Fig. 2). In subgroup analysis, we found significant associations in Asian population (OR = 2.63, 95%CI: 1.36–5.09, *P =* 0.004) but not in South American population (OR = 1.35, 95%CI: 0.69–2.64, *P* = 0.379) (Fig. 3).

### Heterogeneity analysis and quality assessment

Heterogeneity analysis showed a significant high heterogeneity among studies (*I*^*2*^ = 75.6%, *P* < 0.001). In sub-group analysis by ethnicity, heterogeneity was high for Asian (*I*^*2*^ = 79.5%*, P <* 0.001*)* but moderate for South American (*I*^*2*^ = 66.5%*, P* = 0.018). By exploring the potential sources of the heterogeneity, we found that the studies by Erzin et al. [36] and Abadi et al. [30] showed larger effect estimates (OR = 31.07, 95% CI: 8.22–117.52) [36], and OR = 53.65, 95% CI: 11.67–246.69 [30], respectively), as compared with other studies. According to the Newcastle-Ottawa study quality assessment scale, we found that studies with score of 7 showed a significant association (OR = 2.27, 95% CI: 1.34–3.85). However, we didn’t find significant association among studies with score of 6 or 5 (OR = 2.07, 95% CI: 0.90–4.77 and OR = 2.01, 95% CI: 0.79–5.11, respectively) (Figure S1).

### Publication bias and sensibility analysis

Publication bias was evaluated by Begg’s and Egger’s test. The visual inspection of funnel plot revealed that there was no significant evidence of asymmetry distribution. And no significant publication bias was observed based on Begg’s test (*P* = 0.284) or Egger’s test (*P* = 0.288) (Figure S2). The impact of each study on the pooled OR was examined by repeating the meta-analysis while excluding individual study, which confirmed the stability of our results (Figure S3).

## Discussion

To date, numerous studies have assessed the association between *H. pylori babA2* gene and GC risk, but results remained inconsistent. The controversial results of individual studies may be due to relatively small sample size. Meta-analysis is an important approach to pool multiple studies and therefore may result in more precise and robust conclusion. In this meta-analysis, we included 20 studies focusing on *H. pylori babA2* gene and GC risk with a total of 1289 patients and 1081 controls. We found that *H. pylori babA2* gene was significantly associated with risk of GC. There is evidence that *H. pylori* increase the risk of GC development through the sequence of atrophy and metaplasia originate from several studies. Chronic *H. pylori* induced inflammation which can probably lead to loss of normal gastric mucosal, with gastric gland destruction, and replacement by fibrosis [53]. The *H. pylori* strains virulence factors, host and environmental factors are main factors to contribute in clinical infection manifestations [54]. And it is well showed that gene encoding pathogenic *H pylori* factors are involved in GC development and colonization properties [29, 49].

The increased GC risk was also associated with co-expression of *H pylori vacAs1*, *cagA* and *babA2* genes [17, 18, 23, 55]. Furthermore, interaction between host’s immunological defenses and *H pylori* virulence factors may play an important role in the development of GC [56, 57]. It was showed that *bab*A2 as a virulence marker could predict clinical outcome, which was dependent on the geographic origin of the *H. pylori* strains [27].

In our study, sub-group analysis according to geographical areas showed that *H. pylori babA2* was not significantly associated with the risk of GC among South American. Our results are comparable to previous studies from South America [19, 26, 32, 34, 50]. The difference among populations may be due to the small sample size for each population, heterogeneity between studies and geographical factors. In other hand, *H. pylori babA2* gene was closely involved in the risk of GC in Asian population, which was confirmed by original studies from Asian population [29, 30, 36, 38].

Our meta-analysis showed some limitations. Firstly, we didn’t obtain original data, which have limited further evaluation of potential gene-gene and gene-environmental interactions. Secondly, the sample sizes of most included studies are relative small. Thirdly, additional analysis based on other factors, such as age, gender, family history, other virulence factors, environment factors (e.g. alcohol intake, smoking, high BMI) and GC subtypes (e.g. intestinal, diffuse or mixed type), could not be analyzed because of the limited information obtained from included studies. Finally, high heterogeneity among studies indicates that the pooled estimation risk should be interpreted with caution.

## Conclusions

Our results suggest that the presence of *H. pylori* with positive *babA2* gene may contribute to increased risk of GC, especially in Asian population. Studies with large sample size are necessary to further elucidate the interaction among environmental factors, bacterial genotype and host factors on GC risk.

## Supplementary information

**Additional file 1: Figure S1.** Sub-group analysis of the association between *H. pylori babA2* gene and gastric cancer risk according to study quality assessment. **Figure S2.** Funnel plot of case–control studies evaluating the association between *H. pylori babA2* gene and gastric cancer risk. Each point represents a study to indicate an association. **Figure S3.** Influence of the summary OR coefficients on the association between *H*. *pylori babA2* gene and gastric cancer risk.

## Data Availability

The datasets used and/or analysed during the current study are available from the corresponding author on reasonable request.

## Abbreviations

*babA*: Blood-group antigen-binding adhesion
BMI: Body mass index
CI: Confidence interval
GC: Gastric cancer
*H. pylori*: *Helicobacter pylori*
NOS: Newcastle-Ottawa Scale
OR: Odds ratio
