# Supplementary material for: Association of Helicobacter pylori babA2 gene and gastric cancer risk: a meta-analysis
Source: BMC Cancer. 2020 May 24;20:465. doi: 10.1186/s12885-020-06962-7 (PMC7247142; doi:10.1186/s12885-020-06962-7)

**Supplementary File**

**Figure S1. Sub-group analysis of the association between *H. pylori babA2* gene and gastric cancer risk according to study quality assessment.**


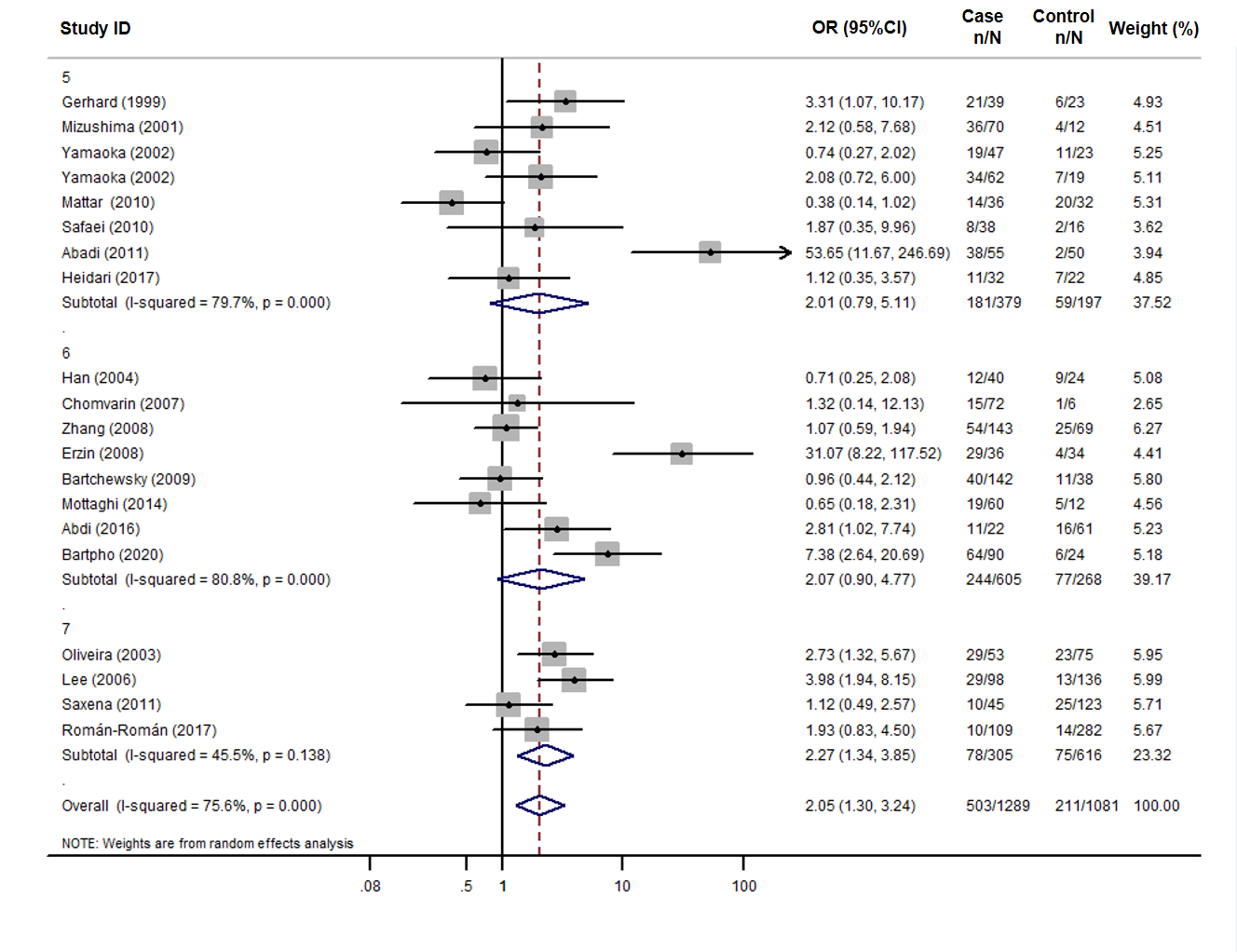


**Figure S2**. **Funnel plot of case–control studies evaluating the association between *H. pylori babA2* gene and gastric cancer risk.** Each point represents a study to indicate an association.

**
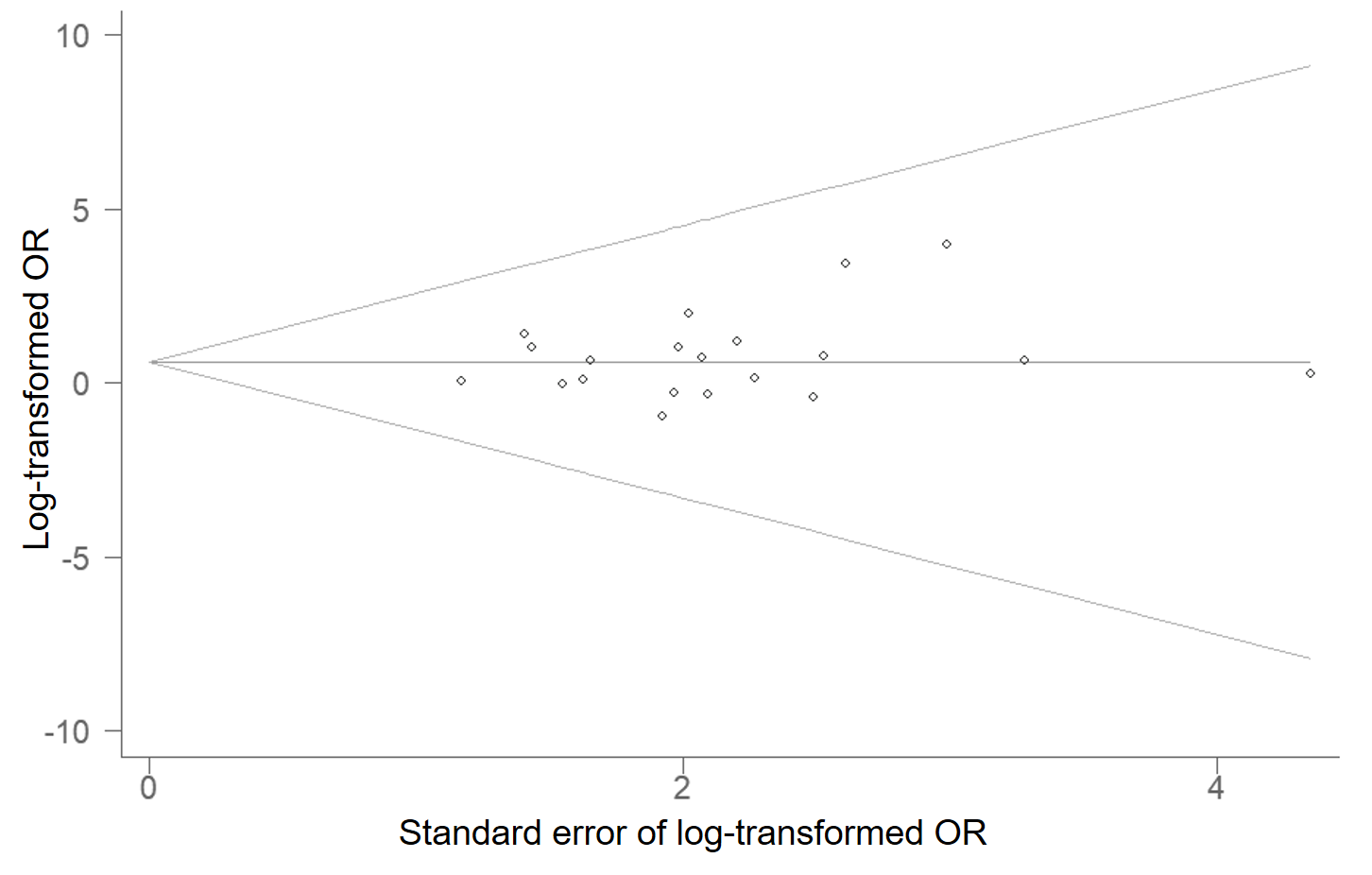
**

**Figure S3**. **Influence of the summary OR coefficients on the association between *H*. *pylori babA2* gene and gastric cancer risk.**


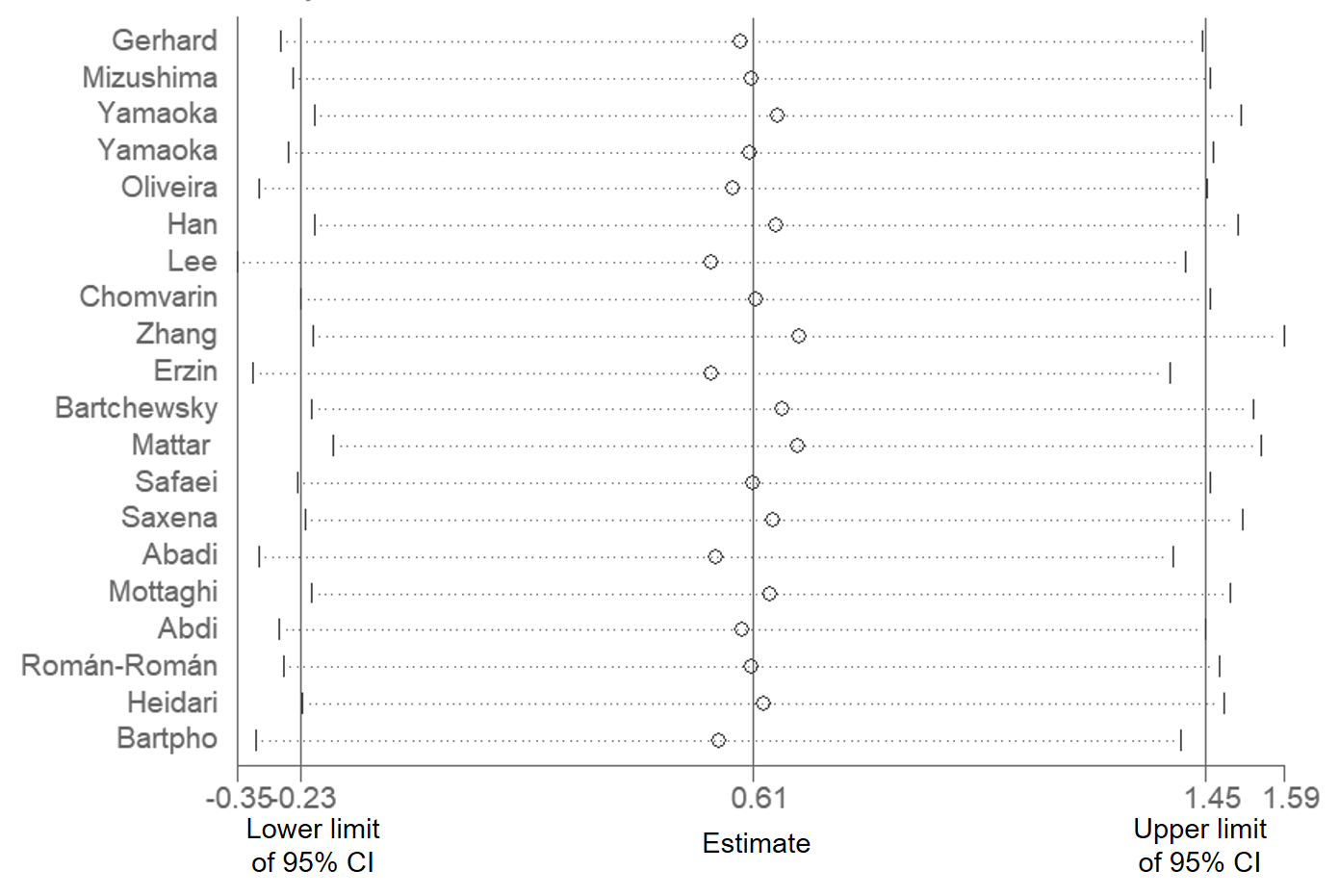

Supplement: Supplementary file 1 — Additional file 1: Figure S1. Sub-group analysis of the association between H. pylori babA2 gene and gastric cancer risk according to study quality assessment. Figure S2. Funnel plot of case–control studies evaluating the association between H. pylori babA2 gene and gastric cancer risk. Each point represents a study to indicate an association. Figure S3. Influence of the summary OR coefficients on the association between H. pylori babA2 gene and gastric cancer risk. [file 12885_2020_6962_MOESM1_ESM.docx]
